# Effect of acupuncture in patients with postprandial distress syndrome: study protocol for a randomized controlled trial

**DOI:** 10.3389/fmed.2025.1521703

**Published:** 2025-02-25

**Authors:** Zhaobo Yan, Xuan Xu, Mailan Liu, Zhimiao MuRong, Huan Zhong, Rong Luo, Haolong He, Weiai Liu, Geshu Du, Mi Liu

**Affiliations:** ^1^School of Acupuncture-Moxibustion, Tuina and Rehabilitation, Hunan University of Chinese Medicine, Changsha, China; ^2^Department of Acupuncture, Moxibustion and Rehabilitation, Changsha Hospital of Traditional Chinese Medicine (Changsha No. 8 Hospital), Changsha, China; ^3^Department of Acupuncture-Moxibustion, The First Affiliated Hospital of Hunan University of Chinese Medicine, Changsha, China; ^4^Department of Acupuncture-Moxibustion, Tuina, and Rehabilitation, The Second Affiliated Hospital of Hunan University of Chinese Medicine, Changsha, China

**Keywords:** acupuncture, clinical trial, functional dyspepsia, postprandial distress syndrome, study protocol

## Abstract

**Background:**

Postprandial distress syndrome (PDS) is the prominent subtype in patients with functional dyspepsia (FD) and currently lacks a satisfactory treatment. Acupuncture has become a promising alternative and complementary therapy for managing FD. However, high-level clinical evidence supporting the use of acupuncture for FD is limited.

**Methods:**

This study is a multicentre, double-dummy, single-blind, randomized, active-controlled trial. Two hundred and one eligible participants will be randomly assigned into three groups: a verum acupuncture plus placebo group, an itopride plus sham acupuncture group, and a sham acupuncture plus placebo group. This study consists of a 1-week screening period, a 4-week treatment period, and a 12-week follow-up period. During the intervention period, participants will receive 12 sessions of verum or sham acupuncture treatment (one session per day, three sessions per week, for 4 weeks) along with 50 mg itopride tablets or 50 mg itopride placebo tablets 3 times a day for 20 days (5 continual days a week for 4 weeks). The response rate (patients who had adequate relief of gastric symptoms will be considered positive responders) and the elimination rate of cardinal symptoms (postprandial fullness and early satiation) are the primary indicators to evaluate the overall acupuncture effect for PDS. Secondary outcome measures will include the Nepean Dyspepsia Symptom Index (NDSI), the short form-Nepean Dyspepsia Life Quality Index (SF-NDLQI), the Hospital Anxiety and Depression Scale (HADS), and related hormone concentrations. Participants’ expectations toward acupuncture treatment will also be assessed, and adverse events will be recorded for safety assessment. All analyses will adhere to an intention-to-treat principle.

**Discussion:**

In conclusion, this trial will determine the efficacy and safety of acupuncture for PDS and provide more high-level evidence to support its application in treating FD.

**Trial registration:**

Identifier [ITMCTR2024000510].

## 1 Introduction

Functional dyspepsia (FD) is a common functional gastrointestinal disorder characterized by upper abdominal discomfort or pain without an identifiable organic cause (1). A recent meta-analysis suggests it impacts 8.4% of adults worldwide (2). The Rome IV criteria categorizes FD into three subtypes: epigastric pain syndrome (EPS), postprandial distress syndrome (PDS), and the overlapping form of EPS/PDS (1). PDS, the most common subtype, presents with meal-related symptoms such as postprandial fullness and early satiety. Epidemiological studies from various countries show that PDS accounts for about 60% of FD cases (3, 4) and is more prevalent in Asian populations compared to Western populations (5). Although not life-threatening, the chronic and recurrent nature of FD symptoms significantly impacts patients’ psychological well-being, quality of life (QOL), and social functioning (6). Furthermore, FD imposes a considerable economic burden on patients (7). Therefore, FD has become a significant public health concern requiring heightened clinical attention and research endeavors.

The effective treatment of FD remains an unsolved clinical challenge. FD is recognized as a complex condition with several factors participating in its development (1, 6). Although drugs such as prokinetic agents, acid suppressants, antidepressants, anti-*Helicobacter pylori* medications, and mucosal protectants have been officially recommended for the treatment of FD, single-drug therapy may not address all of the underlying pathophysiological mechanisms of FD, leading to limited efficacy (8). Additionally, these medications may be limited by side effects (9, 10). Therefore, physicians and patients expect more treatments for FD that are both effective and low-risk.

Notably, acupuncture treatment, a method found in traditional Chinese medicine (TCM), can improve FD symptoms through multiple molecular mechanisms (11). It is currently recognized as a significant complementary and alternative therapy for the management of FD (12). Multiple meta-analyses revealed that acupuncture is more effective than the single use of prokinetic drugs or sham acupuncture in improving digestive symptoms and quality of life in patients with FD (13, 14). Moreover, another system review found that acupuncture combined with Western medicine has a synergistic effect in the treatment of FD while helping to reduce the relapse rate within 3–6 months after treatment (15). Additionally, the evidence has shown that acupuncture can regulate gastrointestinal function by improving gastrointestinal motility and reducing visceral sensitivity, which supports the potential of acupuncture as a therapeutic option for FD (16). However, due to limited high-quality evidence (evidence grade B), a recent consensus statement did not endorse acupuncture for FD, highlighting the need for further high-quality RCTs to clarify its therapeutic potential and role in FD management (8).

Previous clinical trials have compared the therapeutic effects of acupuncture with prokinetic agents in patients with FD, revealing acupuncture as a superior way to prokinetic agents (17, 18). However, these studies were open-lable and lacked blinding, potentially allowing patient preferences for acupuncture to influence clinical outcomes. To obtain more high-quality evidence, we design a new randomized trial; this trial will administer different treatments (acupuncture or the drug itopride) in a double-dummy, single-blind design to objectively evaluate the effectiveness and safety of acupuncture for PDS compared to itopride.

## 2 Methods

### 2.1 Study design

**Figure 1:**
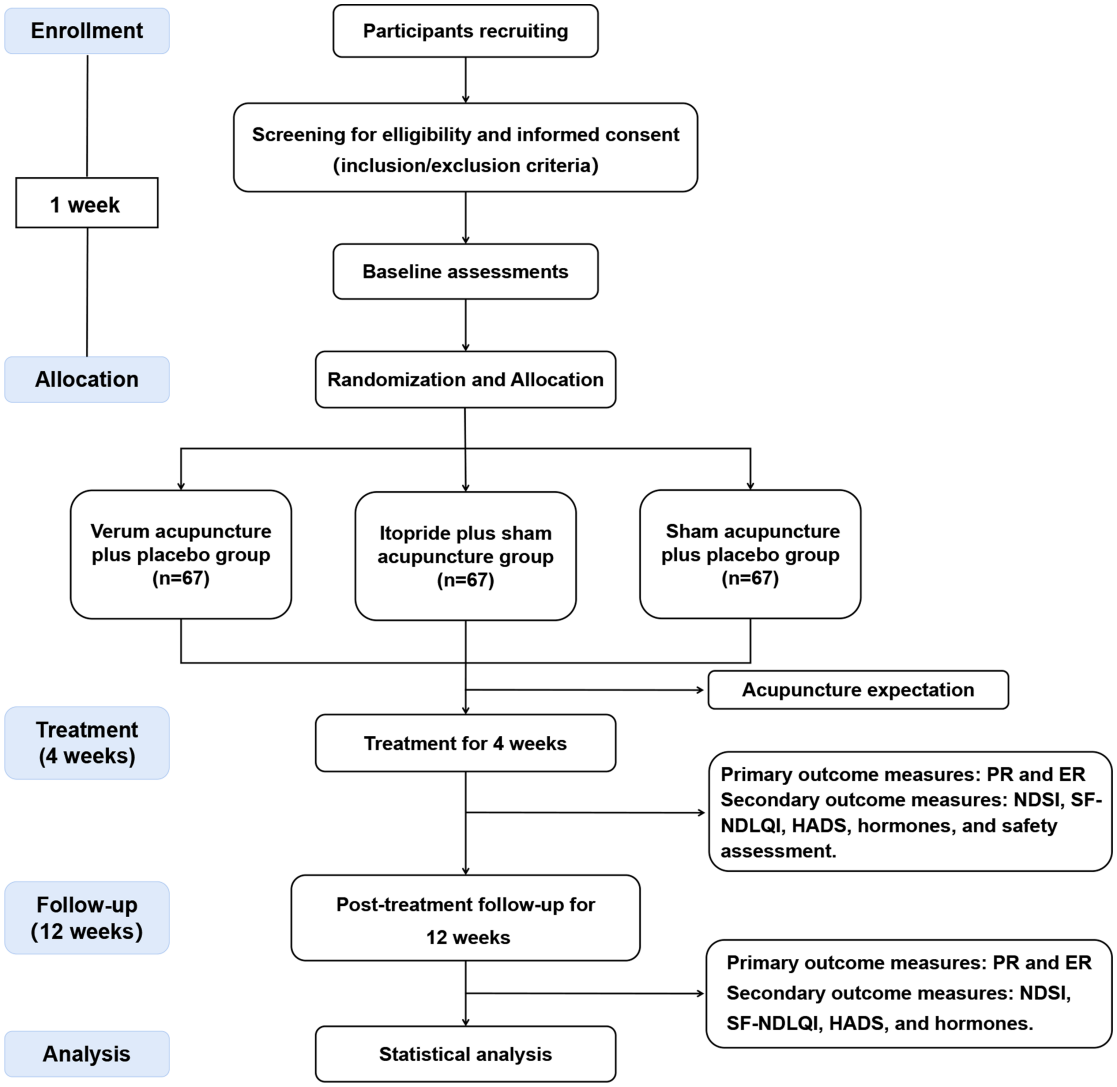
Flowchart of trial procedures. PR, proportion of responders; ER, elimination rate; NDSI, Neapean Dyspepsia Symptom Index; SF-NDLQI, The Short Form-Nepean Despepsia Life Quality Index; HADS, Hospital Anxiety and Depression Scale.

This clinical study is a multicentre, single-blind, double-dummy, randomized, active-controlled trial that compares the efficacy and safety of acupuncture versus itopride in improving upper abdominal discomfort in patients with PDS. The study protocol has been registered with the International Traditional Medicine Clinical Trial Registry (ITMCTR2024000510). It adheres to the Standard Protocol Items: Recommendations for Interventional Trials (SPIRIT) guidelines (Supplementary file 1) (19) as well as the Standards for Reporting Interventions in Controlled Trials of Acupuncture (STRICTA) checklist (Supplementary file 2) (20). The study flowchart is shown in Figure 1.

### 2.2 Study setting and recruitment

This clinical trial will be conducted collaboratively by an academic center and three clinical institutions. Researchers from the Hunan University of Chinese Medicine will form an academic center group responsible for experimental design, data analysis and results dissemination. Recruitment, treatment and data collection will occur at the First Affiliated Hospital of Hunan University of Traditional Chinese Medicine, the Second Affiliated Hospital of Hunan University of Traditional Chinese Medicine, and Changsha Hospital of Traditional Chinese Medicine. Recruitment is scheduled to take place at inpatient and outpatient departments in three clinical institutions, and start in December 2024, with the follow-up assessments of all participants completed by December 2027. Recruitment information will be disseminated through various means such as paper flyers, posters, and social platform like Wechat. Participants interested in this trial will be informed about the purpose and process of the study as well as potential risks and benefits. If the patient decides to participate after understanding this information, they will undergo a clinical screening evaluation conducted by registered TCM practitioners with a minimum of 5 years of clinical experience. Eligible patients will sign an informed consent form, receive baseline assessment, and be randomly assigned.

### 2.3 Eligibility criteria

#### 2.3.1 Inclusion criteria

The study will involve individuals who meet the following criteria: (1) aged 18–60 years and long-term residents of Changsha; (2) diagnosed with FD-PDS according to the Rome IV criteria (21): the individual experiences a sense of fullness and some discomfort in the upper abdomen after normal eating and/or a sense of early satiety which influence the completion of the normal dietary intake occurred several times a week (The past 3 months meet the diagnostic requirements, and symptoms should have been present for a minimum of 6 months prior to diagnosis); (3) no structural abnormalities were detected through upper gastrointestinal endoscopy surveillance; (4) non-refractory FD; (5) patients who are *Helicobacter pylori*-negative or have persistent symptoms after *Helicobacter pylori* eradication for 6 months; (6) Good compliance.

#### 2.3.2 Exclusion criteria

The following individuals will be excluded from the study: (1) use drugs, such as prokinetics, acid suppression, mucosal protectants, or other FD-related agents, within 2 weeks; (2) do not tolerate acupuncture; (3) receive acupuncture treatment within 2 weeks; (4) have gastrointestinal diseases other than PDS; (5) pregnant or lactating women and those attempting conception; (6) patients who are participating in other clinical trials; (7) diagnosed with malignancy or other serious conditions, such as severe heart, liver, lung, or kidney dysfunction, severe autoimmune disease, or endocrine diseases; (8) unable to effectively communicate for any reason, such as severe mental illness, cognitive impairment, or impaired language comprehension.

#### 2.3.3 Dropout exclusion criteria

Patients who discontinue treatment due to any of the following reasons (including but not limited to) will be a dropout case: (1) voluntary withdrawal by participants for any personal reasons; (2) the patient’s symptoms do not improve and gradually worsened after treatment; (3) patients are unreachable due to relocated or changed contact information; (4) patients undergo changes in health status that need a timely manner; (5) patients experience intolerable side effects and are unwilling to continue treatment.

#### 2.3.4 Exit/termination criteria

The exit/termination criteria are as follows: (1) patients do not meet the inclusion criteria and are enrolled mistakenly; (2) patients receive other or additional FD treatments during the study period.

#### 2.3.5 Strategies to improve adherence to interventions

In order to mitigate the impact of dropout rates on research quality, we will enhance participant compliance through several strategies: (1) researchers will regularly contact with participants via WeChat or phone calls to understand their condition and remind them about the schedule of their next visit; (2) we will schedule treatment and evaluation sessions at convenient times to minimize the disruption to participants’ work and daily lives; (3) the evaluation process will be simplified to enhance patient engagement; (4) participants will be reimbursed for treatment expenses and transportation costs to and from the hospital; (5) dedicated staff will be available to address any study-related inquiries raised by participants.

### 2.4 Randomization, allocation, and concealment

A research assistant at each clinical institution will conduct random assignments independently from other study activities. Eligible patients will be randomly assigned into the verum acupuncture plus placebo group, itopride plus sham acupuncture group, and sham acupuncture plus placebo group in a 1:1:1 ratio. A professional statistician will utilize SAS software (v9.3) to produce a list of random numbers stratified by clinical institution using block sizes of 6 or 9. The randomization process will be carried out through an online central randomization system. The randomization number and group assignment provided to each patient by the randomization system will be unique and unchangeable, ensuring fairness in treatment allocation.

### 2.5 Blinding

This study will maintain blinding among patients, observers, and statisticians. However, acupuncturists will not be blinded due to the nature of the acupuncture procedure. Patients will be informed that the study involves two types of acupuncture treatment and two types of drug, which theoretically can improve PDS symptoms. Regardless of their assigned group, patients will receive a combination treatment comprising one type of acupuncture treatment and one type of drug. Sham acupuncture will be performed by using Streitberger needles. Itopride placebo tablets will be indistinguishable from verum itopride tablets in taste and appearance. Each patient will undergo treatment in separate rooms to prevent communication between them. Acupuncturists, who will not be blinded, must refrain from discussing assignment information with participants. In the case the trial goes well, the research center will perform unblinding once the statistical analysis is complete. However, if any emergencies arise, such as life-threatening adverse events (AEs), the academic center will perform urgent unblinding. The monitoring team and ethics committee will then analyze the relationship between patient intervention strategies and AEs.

### 2.6 Intervention

This trial consists of a screening phase (1 week), a treatment phase (4 weeks), and a post-treatment follow-up phase (12 weeks). During the treatment period, participants in the verum acupuncture plus placebo group will receive 12 sessions of verum acupuncture treatment and itopride placebo tablets for 20 days. Patients in the itopride plus sham acupuncture group will take itopride tablets for 20 days and receive 12 sessions of sham acupuncture treatment. Patients in the sham acupuncture plus placebo group will take itopride placebo tablets for 20 days and receive 12 sessions of sham acupuncture treatment. The detailed treatment procedures is as follows:

#### 2.6.1 Verum or sham acupuncture

**Figure 2:**
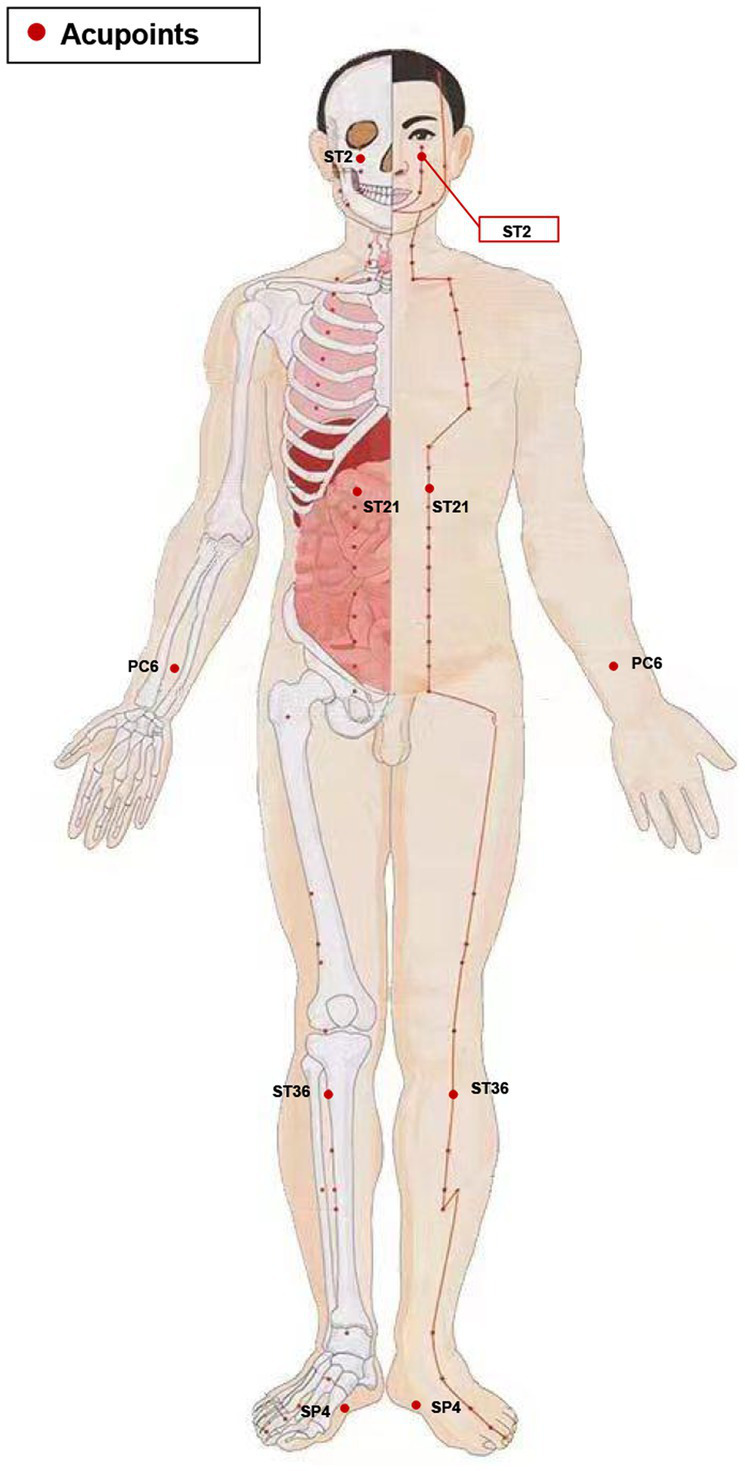
Stimulation points. ST2, Sibai; ST21, Liangmen; ST36, Zusanli; PC6, Neiguan; SP4, Gongsun.

In the verum acupuncture protocol, acupoints of SiBai (ST2), Liangmen (ST21), Zusanli (ST36), Neiguan (PC6), and Gongsun (SP4) were selected based on our prior clinical research on acupuncture for FD (22). The location of all acupoints is followed the Nomenclature and location of acupuncture points per the Chinese National Standard (GB/T22163-2008) and illustrated in Figure 2 and detailed in Table 1. The depth of needle penetration will vary depending on the location of acupoints, refer to Table 1 for details. Before inserting the needle into the skin, acupuncturists will sterilize both the needle (Huatuo brand, Suzhou Medical Supplies Factory, China) and the skin, and an adhesive plaster with plastic rings will be fixed on the skin of acupoints. After the needles penetrate the skin, acupuncturists will manipulated them with lifting, inserting, or twisting movements until patients feel the Deqi sensation. The needles will be stay in place for 30 min, during which they will be manually stimulated every 10 min to elicit the needle sensation.

**Table 1:** Locations and manipulations of acupoints.

Acupoint	Location	Depth of needle penetration in verum acupuncture treatment
Sibai (ST2)	In the face, directly below the pupil, and in the depression of the infraorbital foramen.	Inserted perpendicularly at 0.5 cun with 0.25 mm*25 mm acupuncture needles.
Liangmen (ST21)	In the abdomen, 4 cun above the umbilicus, and 2 cun lateral to the anterior midline.	Inserted perpendicularly at 0.8–1.2 cun with 0.3 mm*40 mm acupuncture needles.
Neiguan (PC6)	In the flexor aspect of the forearm, between the tendons of palmaris longus and flexor carpi radialis, 2 cun above the transverse crease of the wrist.	Inserted perpendicularly at 0.5–0.8 cun with 0.25 mm*25 mm acupuncture needles.
Zusanli (ST36)	In the anterior aspect of the lower leg, 3 cun directly below Dubi (ST35), and one finger-breadth lateral to the anterior border of the tibia.	Inserted perpendicularly at 1–1.5 cun with 0.30 mm * 50 mm acupuncture needles
Gongsun (SP4)	In the anterior and inferior of the base of the first metatarsal bone, and on the dorso-ventral boundary.	Inserted perpendicularly at 0.5–0.8 cun with 0.25 mm*25 mm acupuncture needles

1 cun is defined as the width of the interphalangeal joint of the patient’s thumb. Dubi (ST35) is in the lateral depression of the patellar ligament when the knee is flexed.

**Figure 3:**
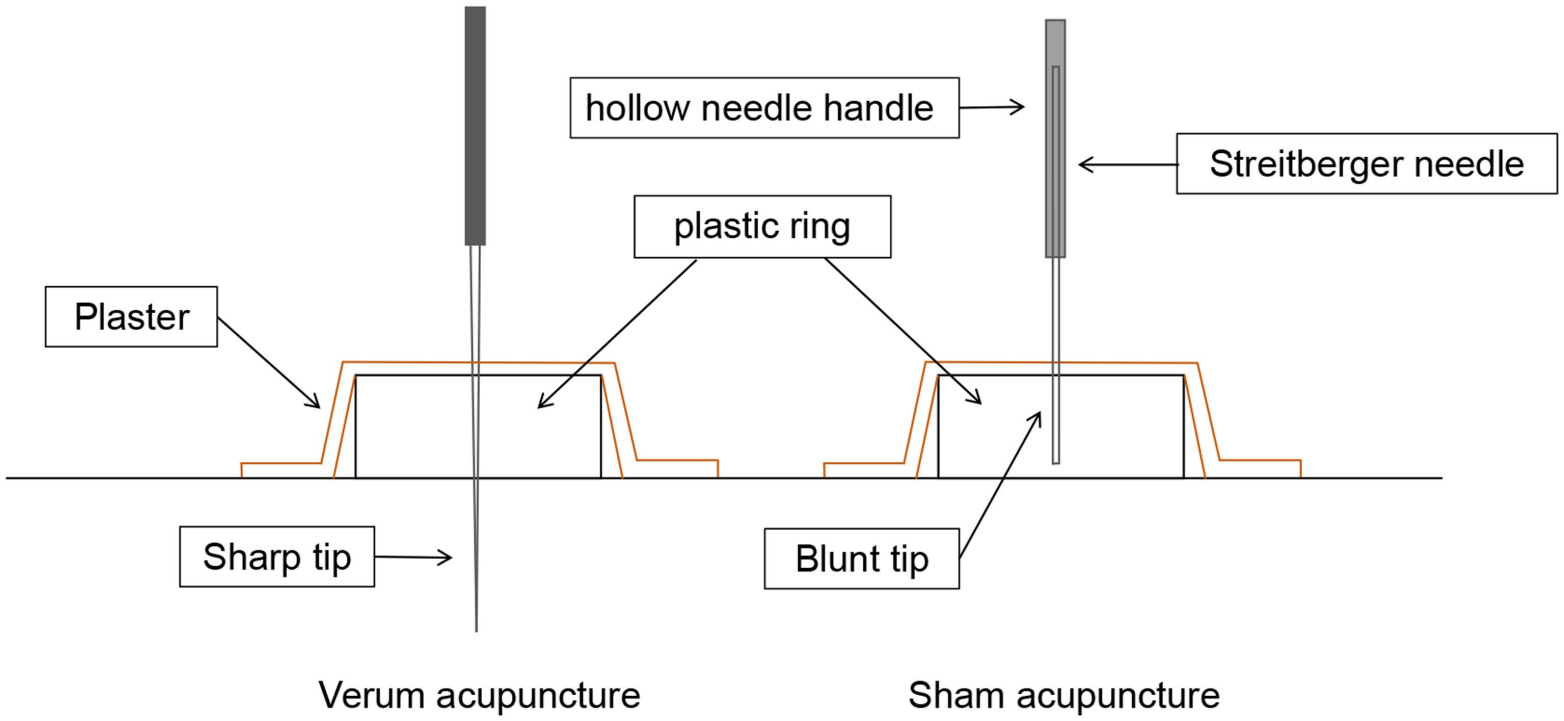
Verum vs. sham acupuncture. Sharp needles are punctured subcutaneously in verum acupuncture, while blunt needles are not punctured subcutaneously in sham acupuncture.

In the sham acupuncture protocol, Streitberger needles will be applied at the same acupoints as those used in the verum acupuncture treatment. The Streitberger needle is a non-penetrating acupuncture placebo device that has been well validated. Although its appearance closely resembles that of real needles, the needle tip is blunt. Furthermore, the Streitberger needle features a special handle that enables the blunt needle to press against the skin surface and retract into the handle rather than penetrate the skin. Nonetheless, a pricking sensation similar to that of actual needle insertion will be generated in the skin at the acupoint during this process. Comprehensive details regarding the verum/sham acupuncture method and needle sizes can be found in Figure 3.

The treatment frequency of verum acupuncture or sham acupuncture will be 3 times a week for 4 consecutive weeks, a total of 12 sessions.

#### 2.6.2 Itopride or placebo

Patients assigned to the itopride plus sham acupuncture group will take 50 mg of itopride orally 3 times a day, 5 days per week, for 4 weeks. Participants in the verum acupuncture plus placebo group and sham acupuncture plus placebo group will receive an itopride placebo that is identical in taste and appearance to actual itopride tablet; they will follow the same frequency and dosage as the itopride plus sham acupuncture group. All patients are instructed to maintain a medication diary to document their usage and return unused medication.

### 2.7 Outcome

Primary outcomes in this study include the proportion of responders (PR) and the elimination rate of the main symptom. The secondary outcomes include changes in symptom score, QOL, mood during the treatment and follow-up, and the participants’ expectancy for acupuncture before treatment, as well as related hormone concentrations. Table 2 display a summary of all measurements. Follow-up for all participants can be completed via social media (WeChat) or phone.

**Table 2:** SPIRIT figure for the schedule of enrollment, interventions, and assessments.

	Study period								
	Enrolment	Allocation	Treatment phase				Follow-up		
Timepoint**	*1w*		*2w*	*3w*	*4w*	*5w*	*9w*	*13w*	*17w*
Enrolment									
Eligibility screen	X								
Informed consent	X								
Allocation		X							
Interventions									
Verum acupuncture plus placebo									
Itopride plus sham acupuncture									
Sham acupuncture plus placebo									
Assessments									
Proportion of responders			X	X	X	X	X	X	X
Elimination rate			X	X	X	X	X	X	X
NDSI		X				X	X	X	X
SF-NDLQI		X				X	X	X	X
HADS		X				X	X	X	X
Acupuncture expectation		X							
Related hormone concentrations		X				X			
Adverse events			X	X	X	X			

SPIRIT, standard protocol items: recommendations for interventional trials; NDSI, the nepean dyspepsia symptom index; SF-NDLQI, the short form - Nepean Dyspepsia Life Quality Index; HADS, Hospital Anxiety and Depression Scale.

#### 2.7.1 Primary outcome

**The proportion of responders (PR)** Patients experiencing sufficient alleviation of stomach discomfort are categorized as positive responders (23). Participants will be requested to evaluate changes in their symptom relief over the preceding 7 days via a 7-point Likert scale. This scale features options such as “extremely worsened,” “worsened,” “slightly worsened,” “no change,” “slightly improved,” “improved,” or “extremely improved,” which correspond to scores ranging from 0 to 6. Those participants who report a relief score of either 5 or 6 points will be deemed positive responders. The measurement of PR will take place at weeks 2, 3, 4, 5, 9, 13, and 17.

**The elimination rate (ER)** ER refers to the proportion of patients whose main symptoms have disappeared. According to Rome IV criteria, we define postprandial fullness and bloating as the main symptoms. Included patients should have at least one of these symptoms. Using a 7-point Likert scale, the main symptoms are considered to have disappeared if the rating drops to 0 after treatment. ER will be measured at weeks 2, 3, 4, 5, 9, 13, and 17.

#### 2.7.2 Secondary outcome

**The Chinese version of the Nepean Dyspepsia Symptom Index (NDSI)** The NDSI is a validated dyspepsia-specific questionnaire that quantifies the severity of 15 upper gastrointestinal symptoms in patients with FD over the past 14 days (24). Each symptom is assessed along three dimensions: frequency, intensity, and degree of interference with daily activities. The higher the NDSI total score, the more severe the dyspeptic symptoms are. During the study, NDSI scores were measured at weeks 1, 5, 9, 13, and 17.

**The Chinese version of Short Form-Nepean Dyspepsia Life Quality Index (SF-NDLQI)** The SF-NDLQI instrument is an FD-specific 10-item questionnaire that assesses the impact of upper gastrointestinal symptoms on QOL across 5 domains: tension, interference with daily activities, eating/drinking, knowledge/control, and work/study (25). Each domain has two items, and each item is scored from 1 to 5, with lower scores better quality of life. In this study, the SF-NDLQI will be administered at weeks 1, 5, 9, 13, and 17.

**The Chinese version of the Hospital Anxiety and Depression Scale (HADS)** The HADS will be employed to evaluate the anxiety and depressive symptoms of patients. This scale consists of 7 items for anxiety (HADS-A) and 7 items for depression (HADS-D), with each item scored from 0 to 3, resulting in a total subscale score range of 0 to 21 (26). Higher scores reflect greater levels of anxiety or depression (27). The HADS has demonstrated strong reliability and validity in screening for anxiety and depression among general medical patients (28). Assessments utilizing the HADS will be conducted at weeks 1, 5, 9, 13, and 17.

**Acupuncture expectation** The Expectation for Treatment Scale (ETS), developed by Jürgen Barth and his colleagues, will be utilized to evaluate patients’ acupuncture expectations before treatment (29). This scale comprises 5 items, such as “I anticipate that the treatment (acupuncture) will alleviate my complaints.” Each item is rated on a scale from 1 to 4, representing partial disagreement, partial agreement, agreement, and definite agreement, respectively. The total score ranges from 5 to 20. A higher total scale score indicates a greater expectation of achieving positive clinical outcomes. The ETS will be measured at week 1.

**Laboratory tests** Serum levels of motilin, gastrin, and ghrelin will be measured using enzyme-linked immunosorbent assays. Fasting venous blood samples (2 mL) will be collected at baseline and after 4 weeks of treatment from all study participants and stored at −80°C until analysis. Laboratory tests will be performed by trained laboratory professionals blinded to treatment assignment.

### 2.8 Safety

AEs are pivotal in evaluating the safety of treatments. All AEs, whether linked to the study treatment (acupuncture or itopride), will be recorded in the case report form (CRF). Each AE will be comprehensively described, including start/stop dates and times, severity, relationship to treatment, actions taken, and the necessity for treatment discontinuation. Researchers will closely monitor all AEs until they are resolved. In the case of serious AEs (e.g., events leading to disability, impaired ability to work, or life-threatening situations), immediate reporting will be made to both the academic center and ethics committee. If any severe AEs associated with acupuncture arise, the ethics committee has the authority to consider suspending the trial.

### 2.9 Data collection and management

Clinical observers will complete a CRF for each participant to record all relevant clinical information. A dedicated data manager will then convert the paper-based CRF data to electronic format, with a secondary check by another research assistant to ensure data accuracy. Participants’ personal information will be anonymized and replaced with code for confidentiality during data collection. All data will be kept confidential, with electronic data secured on a password-protected server and paper data stored in locked cabinets. Access to clinical data will be restricted to authorized researchers only. Raw data will be retained for 5 years. The clinical inspectors/monitors will supervise the data collection and control for data completeness and quality.

### 2.10 Quality control

Before the study begins, the research center will provide standardized training sessions for the entire research team to ensure they follow the study protocol and become familiar with the administration process. Clinical researchers are encouraged to contact the academic center for assistance if any technical or other issues arise at the clinical site. The academic center should promptly provide practical guidance to address these issues. Registered acupuncturists with a minimum of 5 years of experience will perform the acupuncture treatment. These acupuncturists will undergo training and testing before the trial to ensure consistency in their acupuncture operation. A two-level quality inspection system will be established to ensure the implementation and quality of program. Inspectors at each center will conduct monthly quality examinations, while an independent monitoring team will carry out quality monitoring every 3 months. After each quality inspection, quality control reports should be submitted to the research center. Furthermore, any modifications to the research protocol must receive approval from the ethics committee.

### 2.11 Sample size calculation

The sample size for this study was determined based on the responder proportion at the end of a 4-week treatment, with 70.69% for the acupuncture treatment and 55.46% for the itopride treatment, as reported in a previous study (30). The sample size calculation was performed using PASS 15.0 software, with a 1:1 ratio between the verum acupuncture plus placebo group and itopride plus sham acupuncture group. A statistical power of 80% (*β* = 0.2), a one-sided alpha of 0.025 (*α* = 0.025), and a non-inferiority margin of −10% (*Δ* = −10%) were considered in the calculation. The resulting sample size was 57 for each group. Accounting for a 15% dropout rate, each group needed 67 cases, resulting in a total enrollment of 201 cases across three groups.

### 2.12 Statistical analysis

Efficacy analyses will be performed on all patients who were randomized, adhering to the intention-to-treat principle. This approach ensures that all patients are included in the analysis based on the group to which they were initially assigned, regardless of whether they completed the study as intended. Missing data on the primary outcome will be imputed using the multiple imputation method under the missing at-random assumption. If there are significant differences in baseline variables between the two groups, those unbalanced variables will be included as covariates in the analysis of the primary outcome.

We will establish confidence intervals at a 95% level, with a significance level set at 0.05. Continuous data will be reported as mean ± standard deviation (SD) if it is normally distributed. Otherwise, it will be reported as median (interquartile range). For longitudinal continuous data, we will compare groups using repeated-measures analysis of variance (ANOVA), taking into account both group and time-group interactions. Student’s t-test or Wilcoxon rank-sum test will be used for other continuous data. For categorical data, Χ^2^ test or Fisher’s exact test will be applied as appropriate. The strength of linear relationships between variables will be evaluated using linear correlation analysis, and sensitivity analysis will be performed if required. The occurrence of AEs between groups will be summarized using descriptive analysis.

### 2.13 Dissemination policy

We intend to publish the final trial results in medical journals related to functional gastrointestinal disorder or complementary and alternative medicine (CAM) within 2 years of completing the final data collection.

## 3 Discussion

There has been a growing focus in academia on effective treatment for FD in recent years. While some advancements have been achieved in drug treatment for FD (8), the effectiveness is not satisfactory and may come with side effects (9, 10). These problems compel patients to explore other safer and more effective methods in CAM. Acupuncture, a non-pharmacologic therapy in TCM, has been observed to be effective in reliving upper abdominal discomfort and improving QOL in FD patients (13, 14). However, there remains a lack of high-quality clinical studies and evidence supporting the use of acupuncture for FD treatment (8). This study aims to assess the efficacy and safety of acupuncture for FD in a well-controlled trial design, providing evidence to support the clinical application of acupuncture.

For better clinical management, the Rome III Consensus proposed a symptom subtype classification of FD, and Rome IV Consensus adhered to this method (31). Although it remains controversial whether there is any difference in pathogenesis between PDS and EPS (32), choosing a treatment plan based on FD subtypes has become a new trend (33). Evidence shows that PDS is the most prominent subtype, affecting more than 60% of all FD patients (3, 4). Therefore, we selected PDS patients as the subjects of this study and diagnosed FD-PDS patients based on Rome IV criteria. Though multiple mechanisms contribute to PDS development, gastric sensorimotor function disorder has been considered a vital factors (33). Prokinetic agents have been recommended as the first-choice treatment for PDS patients in a Chinese consensus (34). Itopride is a prokinetic agent with a response rate of 55–73% in treating FD and higher drug safety (30, 35, 36). Therefore, we used itopride as a positive drug to evaluate the efficacy of acupuncture in treating PDS, thereby providing more clinical treatment decision-making references.

Five acupoints are selected in this protocol based on findings from clinical and basic research: Sibai (ST2), Zusanli (ST36), Liangmen (ST21), Neiguan (PC6), and Gongsun (SP4). A previous clinical study has shown that acupuncture at above five acupoints can further alleviate discomfort in upper abdominal and improve patients’ QOL based on mosapride treatment (22). ST2, ST36, and ST21 belong to the Yangming stomach meridian of the foot, commonly applied in digestive-system diseases. These three acupoints can regulate the disturbed qi and blood and normalize spleen and stomach function. In particular, acupuncture at ST36 has been proven to improve the gastric function of FD model rats by reducing low-grade inflammation of duodenal mucosal, accelerating gastric emptying, and reducing gut visceral hypersensitivity (37, 38). PC6 and SP4 are both Ba-mai Jiao-hui points. The traditional meridian theory believes that stimulating PC6 and SP4 can positively affect the gastrointestinal tract function and emotional state. Our previous basic studies indicated that acupuncture at PC6 and SP4 could improve gastrointestinal motility disorders, visceral hypersensitivity, and the anxiety and depression-like behavior in rats with FD (39, 40). Therefore, we selected ST2, ST36, ST21, PC6, and SP4 as our acupuncture acupoints.

To our knowledge, this is the first multicenter, randomized, double-dummy, single-blind, active-controlled trial assessing the efficacy and safety of acupuncture for treating PDS. In this trial, we will implement blinding among participants to reduce the potential influence of patient preference for acupuncture on the study results. Furthermore, we plan to adopt stringent quality control measures, blinded outcome assessments, ensured concealed treatment allocation, and intention-to-treat analysis to minimize bias and enhance the credibility of our findings. However, several limitations of this trial should be noted. Although participants and assessors are blinded in this trial, treatment personnel can not be further blinded due to the inherent nature of acupuncture. Therefore, it cannot be ruled out that performance bias influences the results. Second, this trial only includes patients from Changsha City; whether the results can be applied to other populations or regions requires further research. Finally, the proposed acupuncture regimen is standardized dose not account for individual differences among patients, which may limit the efficacy of the acupuncture treatment.

To summarize, the outcome of this study aims to validate the difference of effectiveness and safety between acupuncture and Itopride in treating PDS. These results will serve as a dependable foundation to endorse the practical use of acupuncture in managing FD.

## Trial status

This clinical trial is in progress. Recruitment is scheduled to begin in December 2024 and be completed by December 2027.
